# Laser nanoprinting of 3D nonlinear holograms beyond 25000 pixels-per-inch for inter-wavelength-band information processing

**DOI:** 10.1038/s41467-023-41350-2

**Published:** 2023-09-08

**Authors:** Pengcheng Chen, Xiaoyi Xu, Tianxin Wang, Chao Zhou, Dunzhao Wei, Jianan Ma, Junjie Guo, Xuejing Cui, Xiaoyan Cheng, Chenzhu Xie, Shuang Zhang, Shining Zhu, Min Xiao, Yong Zhang

**Affiliations:** 1 National Laboratory of Solid State Microstructures, College of Engineering and Applied Sciences, School of Physics, and Collaborative Innovation Center of Advanced Microstructures, Nanjing University, Nanjing, 210093 China; 2 School of Physics, Sun Yat-sen University, Guangzhou, 510275 China; 3 Department of Physics, The University of Hong Kong, Hong Kong, China; 4 Department of Electrical and Electronic Engineering, University of Hong Kong, Hong Kong, China; 5 Department of Physics, University of Arkansas, Fayetteville, AR 72701 USA

**Keywords:** Nonlinear optics, Nanophotonics and plasmonics

## Abstract

Nonlinear optics provides a means to bridge between different electromagnetic frequencies, enabling communication between visible, infrared, and terahertz bands through *χ*^(2)^ and higher-order nonlinear optical processes. However, precisely modulating nonlinear optical waves in 3D space remains a significant challenge, severely limiting the ability to directly manipulate optical information across different wavelength bands. Here, we propose and experimentally demonstrate a three-dimensional (3D) *χ*^(2)^-super-pixel hologram with nanometer resolution in lithium niobate crystals, capable of performing advanced processing tasks. In our design, each pixel consists of properly arranged nanodomain structures capable of completely and dynamically manipulating the complex-amplitude of nonlinear waves. Fabricated by femtosecond laser writing, the nonlinear hologram features a pixel diameter of 500 nm and a pixel density of approximately 25000 pixels-per-inch (PPI), reaching far beyond the state of the art. In our experiments, we successfully demonstrate the novel functions of the hologram to process near-infrared (NIR) information at visible wavelengths, including dynamic 3D nonlinear holographic imaging and frequency-up-converted image recognition. Our scheme provides a promising nano-optic platform for high-capacity optical storage and multi-functional information processing across different wavelength ranges.

## Abstract (web summary)

By using femtosecond laser writing technique, the research group in Nanjing University has fabricated 3D nano-resolution nonlinear holograms in lithium niobate crystals, which is capable of processing optical information across the wavelength gaps.

## Introduction

Nonlinear optics is capable of connecting the electromagnetic waves of different bands, which has been extensively investigated for laser frequency conversion and quantum light generation^1,2^. For instance, it is feasible to convert near-infrared (NIR) light to the visible band through second-harmonic generation (SHG) in nonlinear crystals including beta barium borate (BBO), potassium titanyl phosphate (KTP), and lithium niobate (LN). Over the past few decades, the performances of nonlinear optic devices have significantly improved through the introduction of nonlinear microstructures^3–14^. The milestone theory of quasi-phase matching (QPM) utilizes periodic *χ*^(2)^ microstructures to enhance the conversion efficiency of nonlinear optical processes^15^, which has led to advancements in multi-color laser^16,17^, frequency comb^18,19^, and quantum correlation^20,21^. To fully release the potential of nonlinear optics for advanced functions that surpass the wavelength gaps, it is essential to develop a way to manipulate nonlinear optical waves with further improved efficiency and precision.

Holography has been proven to be an important method for optical information processing. Traditionally, it records the amplitude and phase information of a light field in a hologram for data storage^22,23^. Since the invention of computer-generated hologram (CGHs)^24^, its function has been significantly expanded to accomplish complex tasks such as 3D display^25,26^, virtual reality^27–29^, and optical neural network^30–32^. By utilizing various degrees of freedom of light, such as the amplitude, phase, polarization, and orbital angular momentum, as information channels, the capacity of present holographic technique has been considerably enhanced^33–37^.

In recent years, nonlinear hologram has emerged as a promising platform to promote the applications of nonlinear optics^38–50^. Compared to its linear counterpart, a nonlinear hologram records information in the spatial distribution of its nonlinear coefficients such as *χ*^(2)^. While a nonlinear hologram appears a homogeneous material in the linear optical regime, its holographic image can only be revealed at the newly-generated optical frequencies through nonlinear measurement. This feature can be utilized for encryption storage^51–57^. By properly designing *χ*^(2)^ structures, nonlinear hologram can perform nonlinear holographic imaging. However, the functions of previous nonlinear holograms with 2D microstructures are mainly focused on the phase modulation of nonlinear waves for reconstructing static plane images. The recent developments in femtosecond laser writing technique make it possible to fabricate 3D *χ*^(2)^ structures (i.e., nonlinear photonic crystals (NPCs)) at nanoscale resolution^58^, which provides a powerful platform to realize multi-functional nonlinear holograms.

In this work, we propose and experimentally demonstrate a 3D nano-resolution *χ*^(2)^-super-pixel hologram for inter-wavelength-band information processing including 3D dynamic nonlinear holography and frequency-up-converted image recognition. In our design, the super-pixels consist of various *χ*^(2)^ structure (i.e., nanodomains) that are capable of fully controlling the phase and amplitude of nonlinear waves. Taking advantage of 3D laser writing, we can arrange these functional nanodomains along the depth direction of hologram, making it possible to realize high in-plane spatial resolution. In experiment, the super-pixel diameter is 500 nm and the pixels-per-inch (PPI) is beyond 25,000. The length of each super-pixel (along the depth direction) is several microns (see “Methods”). Importantly, for a given *χ*^(2)^-super-pixel hologram, the output nonlinear fields can be dynamically tuned by changing the input wavelength, light polarization, or crystal temperature.

## Results

### Design of *χ*^(2)^-super-pixel hologram

**Fig. 1:**
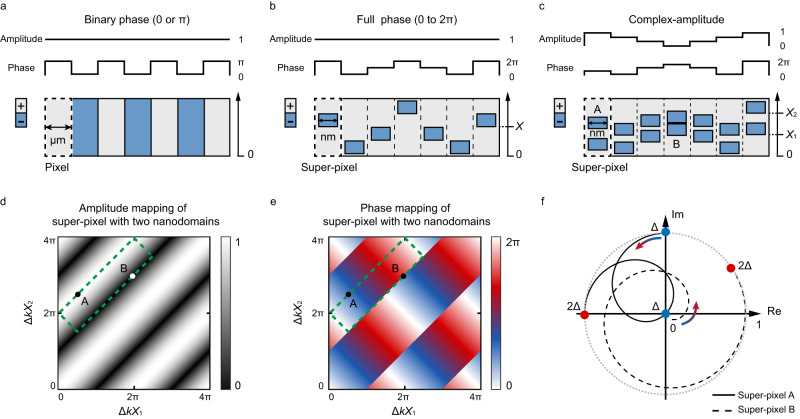
Principle of super pixel. **a**–**c** Three schemes for NPC-based nonlinear hologram. **a** The traditional binary phase modulation of nonlinear waves, which has been widely applied in previous nonlinear holography works. In (**b**), a negative domain is embedded inside the positive ones. This type of super pixel is capable of fully controlling the phase of nonlinear waves. In (**c**), two negative domains are properly arranged inside each super pixel, which can be utilized to realize the complete complex-amplitude modulations of nonlinear waves. **d** and **e** show the dependences of the amplitude and phase of nonlinear wave on the value of $\Delta kX$, respectively. The dotted lines indicate the parameters used in this work. **f** The dynamic evolutions of the complex-amplitude from super pixels A (solid line) and B (dotted line) as increasing the value of $\Delta k$ from Δ to 2Δ. The initial amplitudes and phases of pixels A and B are marked in (**d**) and (**e**). See “Methods” and Supplementary Fig. 3 for details.

Consider a traditional nonlinear holographic configuration in 2D NPCs (Fig. 1a). The pixel of nonlinear hologram refers to a positive (or negative) domain, which has a nonlinear coefficient of +*χ*^(2)^ (or −*χ*^(2)^). The pixel diameter is typically several microns due to the limitations of the traditional electrically-poling techniques. The nonlinear waves generated in these domains are binary-phase modulated, i.e., carrying 0 or *π* phase (Fig. 1a). Correspondingly, designs of traditional nonlinear holograms are mainly based on binary CGH theory. To improve the performance of nonlinear holograms, it is necessary to develop more powerful techniques to fully control the phase and amplitude of nonlinear waves at higher resolution.

Femtosecond laser writing of 3D nanodomains provides a useful solution. For instance, one can fabricate super-pixels consisting of nanoscale domain structures to upgrade the functionalities and resolution of nonlinear hologram. Figure 1b shows an example, in which a super-pixel consists of a negative nanodomain embedded inside the positive ones. The position of the negative domain is defined by *X*. Here we take the sum frequency (SF) generation as example. When two fundamental waves (*ω*_1_ and *ω*_2_) are incident along the *x* axis of NPC sample, the complex-amplitude of the SF wave (*ω*_3_ = *ω*_1_ + *ω*_2_) produced at such super-pixel satisfies (see “Methods”)

1 $${A}_{3}\propto \exp (i\Delta kX),$$

where $\Delta kX=({k}_{3}-{k}_{1}-{k}_{2})X$ is the extra phase introduced by the nanodomain structure inside the super-pixel, which can be continuously tuned by varying *X*. Here, *k*_1_, *k*_2_ and *k*_3_ are the wave vectors of two incident fundamental waves and SF waves, respectively. Clearly, one can use Eq. (1) to compose a fully phase-modulated nonlinear hologram.

In addition, one can utilize a pair of negative domains within each supercell to realize the complete control of the phase and amplitude of SF waves. Figure 1c shows the schematic configuration. Here, two negative nanodomains have the same dimensions and their positions are defined by *X*_1_ and *X*_2_, respectively. The SF complex-amplitude can be written as (see “Methods”)

2 $${A}_{3}\propto \,\cos \Delta k\frac{{X}_{1}-{X}_{2}}{2}\exp \left(i\Delta k\frac{{X}_{1}+{X}_{2}}{2}\right).$$

For given input fundamental beams, Δ*k* is normally fixed, and the amplitude and phase of SF wave are then decided by $({X}_{1}-{X}_{2})/2$ and $({X}_{1}+{X}_{2})/2$, respectively. By varying the values of *X*_1_ and *X*_2_, one can realize the complete control of the phase (0–2*π*, Fig. 1e) and amplitude (0–1, Fig. 1d) of the SF waves.

### 3D field encryption and reconstruction

**Fig. 2:**
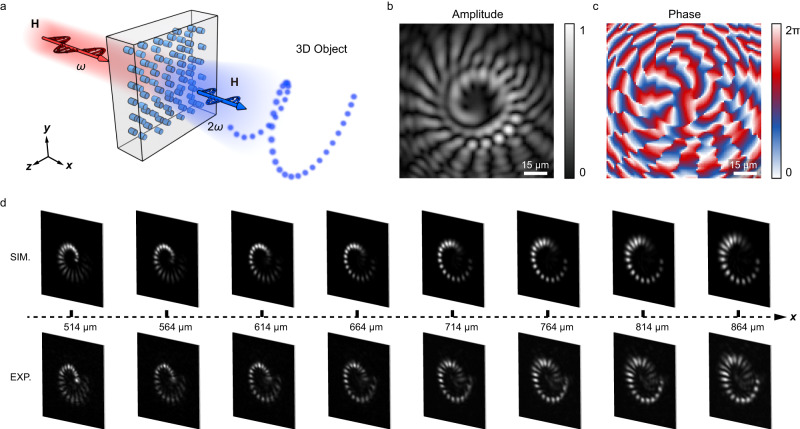
The generation of a 3D spiral line at SH wave. **a** The schematic diagram of nonlinear 3D information reconstruction. The *z*-axis is along the optical axis of LiNbO_3_ crystal. **b** and **c** show the amplitude and phase distributions of nonlinear hologram, respectively. **d** The experimental (bottom) and simulated (up) results. The SH patterns at various distances well present a 3D spiral line.

In experiment, we first demonstrate the use of such nonlinear hologram in 3D field encryption and reconstruction. Figure 2 shows the generation of 3D spiral line at second harmonic (SH) wave. High-resolution complex-amplitude nonlinear hologram is designed through the direct Fresnel transform of the 3D object. The calculated amplitude and phase distributions of the hologram are shown in Fig. 2b c, respectively. By using Eq. (2), one can design the corresponding ${\chi }^{(2)}$ structure, i.e., deducing the values of *X*_1_ and *X*_2_ in each pixel (see “Methods”). The sample is fabricated by using the laser writing technique in ref. ^58^. The super-pixel diameter is 500 nm and the interval is 500 nm. In the SH generation experiment, the light source is a femtosecond laser operating at a wavelength of 840 nm. The SH patterns at propagation distances from 514 μm to 864 μm are shown in Fig. 2d, which show a clear trajectory of a 3D spiral line. The numerical simulations agree well with the experimental results (Fig. 2d). The 3D SH spiral line produced in this way is speckle-free, benefiting from the complete complex-amplitude modulation of SH waves. Notably, the 3D field is not present in the fundamental wavelength channel, a feature that can be very useful for security encryption (see Supplementary Note 1).

We further show that our configuration is capable of dynamic 3D field reconstruction. As shown in Eq. (2), the generation of SH wave is related to Δ*k*. In experiment, Δ*k* can be readily tuned by changing the wavelength or polarization of the input light, or modulating the crystal temperature. This provides a convenient way to dynamically tune the complex amplitude of SH wave in a fixed *χ*^(2)^ structure. In numerical simulations, we choose two different super-pixels (A and B in Fig. 1c) as examples. Their performances evolve along different curves as the value of Δ*k* is gradually increased from $\Delta$ to 2$\Delta$ (Fig. 1f). At the end, the phase of pixel A is doubled while the amplitude of pixel B increases from 0 to 1, which shows a wide tuning range under our configuration. In Supplementary Fig. 3 and Table 1, we show the full access to the complex-amplitude modulation of nonlinear waves.

**Fig. 3:**
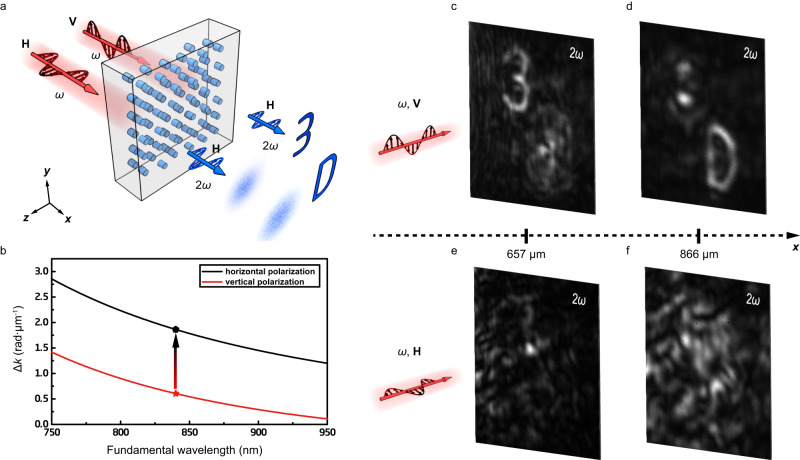
3D information encryption through polarization-dependent nonlinear hologram. **a** In our scheme, 3D information is encrypted into the vertical polarization channel. **b** The wavelength-dependence of $\Delta k$ at horizontal (black) and vertical (red) polarizations. **c**–**f** The experimental results at SH waves. By using a vertically-polarized input light, one can reconstruct two letters 3 and D at distances of 657 μm and 866 μm, respectively. In comparison, only SH speckles present under the illumination of a horizontally polarized fundamental light.

We design two experiments to show dynamic performance of nonlinear holograms. First, we utilize the polarization of the input light to demonstrate encryption storage of 3D information (Fig. 3a). In theoretical design, we first calculate Δ*k* with different light polarizations (Fig. 3b). Because of the birefringence of LiNbO_3_ crystal, the value of Δ*k* at a fundamental wavelength of 840 nm is tripled when the polarization of the input light is switched from vertical to horizontal. Such difference is sufficient for designing a nonlinear hologram for 3D information encryption in one polarization channel, while the information is indistinguishable in the other (Supplementary Fig. 4). In the experiment, the 3D target image includes two letters (3 and D) at different propagation depths of SH wave, which are stored in the vertical polarization channel. Figure 3c–f shows the experimental results. Under the illumination of a vertically-polarized fundamental beam, the SH field presents two clear letters—3 and D at propagation distances of 657 μm and 866 μm, respectively. However, with a horizontal polarization, only random SH patterns can be observed, from which one can hardly identify the original information. Hence our configuration is capable of encrypting 3D information by using the polarization dimension of light.

**Fig. 4:**
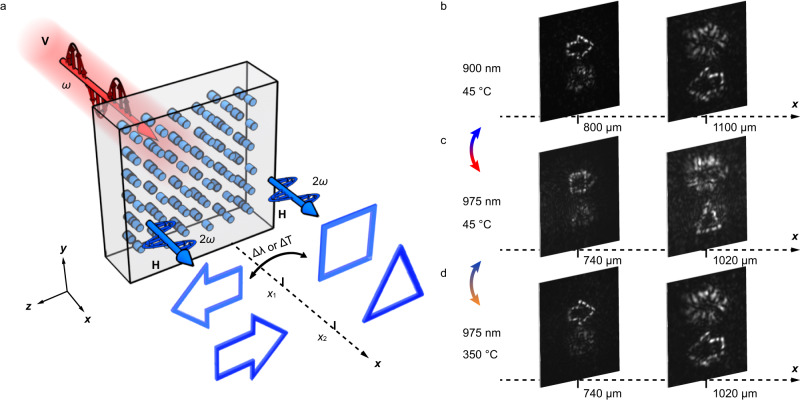
Dynamic 3D information reconstruction based on wavelength-temperature joint modulation. **a** In our scheme, the dynamical projections of 3D objects at SH wave are realized by tuning the crystal temperature and the input wavelength. The incident fundamental light is vertical polarization and output SH wave is the horizontally polarization. The *z*-axis is along the optical axis of LiNbO_3_ crystal. **b**–**d** The experimental results. At the initial state (i.e., 900 nm and 45 °C), two arrows present at different propagation depths of SH waves. In the middle state (i.e., 975 nm and 45 °C), the SH patterns become a square and a triangle at different distances. At the end state (i.e., 975 nm and 350 °C), two arrows present again except that their locations are different from those in (**b**).

Next, we show the wavelength-temperature joint modulation for dynamic switch of 3D information. One can gradually tune the light wavelength or the sample temperature to realize continuous modulation of Δ*k*. The hologram design is based on the iterative Fourier transform algorithm (see “Methods”). The experimental configuration is shown in Fig. 4a and the experimental results are shown in Fig. 4b–d. We first set the incident wavelength to 900 nm and the crystal temperature at 45 °C, two SH arrows with opposite directions appear at 800 μm and 1100 μm, respectively, away from the sample (Fig. 4b). When we change the incident wavelength to 975 nm and maintain the crystal temperature, the output SH image becomes a square and a triangle locating at distances of 740 μm and 1020 μm, respectively (Fig. 4c). Finally, with the increase of the temperature to 350 °C, two SH arrows reappear (Fig. 4d) but a different location from that in Fig. 4b. Note that the SH patterns in Fig. 4b–d correspond to different values of Δ*k*. Our scheme is also capable of dynamic nonlinear beam shaping. In Supplementary Note 2, we present the dynamic conversions between three different orbital angular momentum modes at SH waves.

### Frequency up-converted optical image processing

Certain applications require the detection of an object at infrared and terahertz bands. However, it is generally difficult to directly manipulate these light fields due to the lack of efficient optical elements in these bands. In this context, we present a solution that involves converting invisible information in these bands to visible wavelengths by using our 3D nonlinear hologram.

**Fig. 5:**
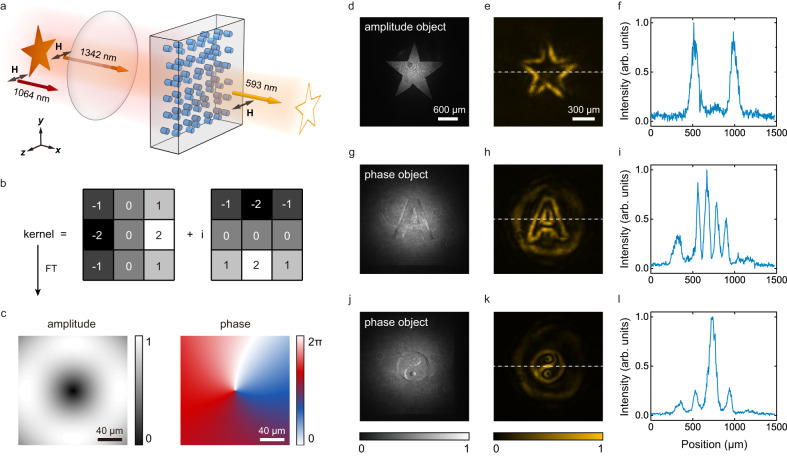
Frequency up-conversion edge enhancement. **a** The experimental diagram. A 1342 nm light (carrying the object information) and a 1064 nm light are frequency-summed in the designed nonlinear hologram. The output SF light at 593 nm presents the edge-enhanced image. **b** presents the convolution kernel, which is a complex-value matrix including two orthogonal differentiation operators. **c** The amplitude and phase of *χ*^(2)^-super-pixel hologram that works as a spatial frequency filter at the SF wavelength, which realizes the convolution function together with two Fourier lenses. **d**, **g**, **j** The NIR images of three objects at 1342 nm. **e**, **h**, **k** The experimental results of frequency up-conversion edge enhancement at 593 nm, in which the dotted lines present good contrasts as shown in (**f**), (**i**), and (**l**), respectively.

As a first example, we demonstrate frequency-up-converted edge enhancement of an NIR image (Fig. 5a). The edge enhancement in linear optics is generally realized through a convolution operation between the original image and a complex-valued kernel (Fig. 5b). In our experiment, the complex-value *χ*^(2)^-super-pixel hologram is designed as a spatial frequency filter. By placing it between two Fourier lenses, one can implement a nonlinear convolution working at the up-converted optical frequency (Fig. 5c). By using such 3D nonlinear hologram, one can readily achieve frequency-up-converted edge enhancement of an amplitude or a phase object. In experiment, we use one amplitude object and two phase objects as examples. We first record the NIR images by illuminating the objects with a 1342 nm beam. The NIR image of the amplitude object is shown in Fig. 5d. For the phase objects, we obtain their linear interference patterns with a Gaussian beam (Fig. 5g, j), in which one can hardly recognize the details. Next, we perform frequency-up-converted edge enhancement by shining an extra 1064 nm beam together with the 1342 nm beam onto the prepared nonlinear hologram. The object information is hence transferred to the up-converted SF light at 593 nm, resulting in enhanced object edges as shown in Fig. 5e, h, k. One can clearly observe the edge details due to the improved contrast (Fig. 5f, i, l).

**Fig. 6:**
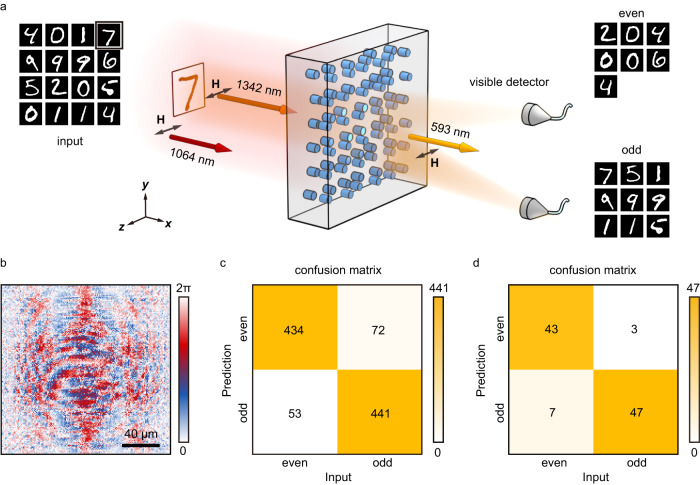
Frequency up-conversion parity classifier of handwritten numbers. **a** The experimental diagram. We use a 1342 nm light to illuminate the number and combine it with a 1064 nm light. Then, we input these NIR lights on the designed nonlinear hologram. The output SF light at 593 nm is focused onto different positions according to the number parity. **b** The phase distribution of nonlinear hologram. **c** and **d** shows the confusion matrices after testing 1000 and 100 handwritten numbers in simulation and experiment, respectively. The diagonal and off-diagonal elements in (**c**) and (**d**) represent the correct and incorrect predictions, respectively.

We further use a *χ*^(2)^-super-pixel hologram to construct a nonlinear diffractive optical neural networks (DONN)^59^ for classifying odd and even numbers carried by an NIR beam at an up-converted frequency (Fig. 6a). Such nonlinear DONN is trained based on the error backpropagation and gradient descent method. Notably, its function is realized by manipulating the frequency-up-converted light field through the optimized nonlinear hologram (see “Methods”). In experiment, a 1342 nm beam illuminates a phase-type handwritten number. Then, it passes through the nonlinear hologram along with a 1064 nm beam (Fig. 6b). The resulting frequency up-converted signal (i.e., the SF light) is focused into two different positions on the screen according to the number parity. In numerical simulations, we achieve an accuracy of 87.5% after classifying 1000 handwritten numbers (Fig. 6c). In experiment, we use 100 handwritten numbers to test our system. The classification accuracy is 90%, which is slightly higher than the value in the simulations due to the use of different data sets (Fig. 6d).

## Discussion

In this Article, we have experimentally demonstrated 3D nonlinear hologram and its unique functions in inter-wavelength-band information processing, which expands the ability of nonlinear optics from traditional frequency conversion to advanced optical processing tasks. Taking advantage of the recent laser writing technique, we are able to prepare high-quality 3D nonlinear holograms beyond the state-of-the-art. Not only can the hologram resolution be substantially improved, but the functionality of each pixel can also be expanded from the traditional binary phase modulation to the complete and dynamic control of the amplitude and phase of nonlinear waves. Correspondingly, many previous technical limitations for nonlinear optic applications can be removed. Besides the demonstrated dynamic 3D nonlinear holography and frequency-up-converted image recognition, our ultra-high-resolution 3D nonlinear holograms can also be utilized for other fascinating applications such as multiple-color display, nonlinear optical computing, and quantum holography.

To meet the requirements of advanced optical information processing, the major challenge is to increase the dimensions of 3D nonlinear holograms. The precision and stability of laser writing system should be improved for large-scale nano-fabrication. Also, it is important to develop parallel processing techniques for fast fabrication of 3D nonlinear holograms. Another technical issue is the non-uniform structure along the depth direction, which may be resolved by pre-shaping the laser writing beam through spatial light modulator.

In comparison to previous planar nonlinear holograms, 3D nonlinear hologram has potential advantages in capacity and conversion efficiency. Because of the extra modulation dimension of *χ*^(2)^, one can arrange more pixels in 3D nonlinear hologram to achieve high capacity and multiple functions. In addition, 3D nonlinear hologram is capable of satisfying the phase matching condition for high conversion efficiency, which is critical to realize practical nonlinear optic devices. The proposed nano-resolution 3D nonlinear hologram is naturally capable of performing complicated inter-wavelength-band processing tasks that require high capacity, high fidelity, and high efficiency. 3D nonlinear hologram is a powerful platform for classic and quantum optical information processing in future.

## Methods

### The theory of ${\chi }^{(2)}$ super-pixel

First, we consider the ${\chi }^{(2)}$ super-pixel with one negative domain inside it (Fig. 1b). Here, we set the lengths of negative domain and super-pixel to be *l* and *h*, respectively. The ${\chi }^{(2)}$ distribution along the *x* direction is given by

3 $${\chi }^{(2)}(x)={\chi }^{(2)}\left[1-2s(x)\right]$$

with *s* is structure function defined by

4 $$s(x)=\left\{\begin{array}{c}1,\\ 0,\end{array}\right.\begin{array}{c}\,X \, < \, x \, < \, X+l \hfill \\ 0\le x\le X\,{{{{{{\rm{and}}}}}}}\,X+l\le x\le h\end{array}.$$

Here, *X* is the position of the negative domain. When *s* = 0 (or 1), it corresponds a nonlinear coefficient of ${\chi }^{(2)}$ (or –${\chi }^{(2)}$). According to nonlinear wave coupled-equation, the generated SF wave can be described by^60^

5 $${A}_{3}={\int }_{\!\!\!0}^{h}\frac{i{\omega }_{3}}{{n}_{3}c}{\chi }^{(2)}(x){A}_{1}{A}_{2}\exp \left(i\Delta {kx}\right){dx}.$$

Here, ${A}_{1},{A}_{2}\,{{{{{\rm{and}}}}}}\,{A}_{3}$ are the complex-amplitudes of two incident waves and the generated SF wave, respectively. ${n}_{3}$ is the refractive index of the SF wave, $\Delta k={k}_{3}-{k}_{1}-{k}_{2}$ with *k*_1_, *k*_2_ and *k*_3_ being the wave vectors of two incident waves and the SF wave, respectively, and *c* is speed of light. By substituting Eq. (3) into Eq. (5), one can obtain

6 $${A}_{3}	={\int }_{\!\!\!0}^{h}\frac{i{\omega }_{3}}{{n}_{3}c}{\chi }^{\left(2\right)}{A}_{1}{A}_{2}\exp \left(i\Delta {kx}\right){dx} -{\int }_{\!\!\!X}^{X+l}\frac{2i{\omega }_{3}}{{n}_{3}c}{\chi }^{\left(2\right)}{A}_{1}{A}_{2}\exp \left(i\Delta {kx}\right){dx}\\ 	={C}_{0}+C\exp \left(i\Delta {kX}\right)$$

Here, ${C}_{0}=\frac{i{\omega }_{3}}{{n}_{3}c}{\chi }^{\left(2\right)}{A}_{1}{A}_{2}\frac{\exp \left(i\Delta {kh}\right)-1}{i\Delta k}$ and $C=-\frac{2i{\omega }_{3}}{{n}_{3}c}{\chi }^{\left(2\right)}{A}_{1}{A}_{2}\frac{\exp \left(i\Delta {kl}\right)-1}{i\Delta k}$. ${C}_{0}$ represents the background, which can be avoided by adding a blazing grating into the hologram. Since *C*_0_ and *C* are constants for given input lights, one can obtain ${A}_{3}\propto \exp \left(i\Delta {kX}\right)$.

Next, we analyze the super-pixel with two negative domains inside it (Fig. 1c). The ${\chi }^{(2)}$ distribution can be written as

7 $${\chi }^{(2)}(x)={\chi }^{(2)}\left[1-2{s}_{1}\left(x\right)-2{s}_{2}\left(x\right)\right].$$

*s*_*1*_ and *s*_*2*_ (defining the two negative domains) are expressed by

8 $$\,{s}_{1}(x)=\left\{\begin{array}{c}1,\\ 0,\end{array}\right.\begin{array}{c}\,{X}_{1} \, < \, x \, < \, {X}_{1}+l \hfill \\ 0\le x\le {X}_{1}\,{{{{{{\rm{and}}}}}}}\,{X}_{1}+l\le x\le h\end{array}\,,$$

9 $${s}_{2}(x)=\left\{\begin{array}{c}1,\\ 0,\end{array}\right.\begin{array}{c}\,{X}_{2} \, < \, x \, < \, {X}_{2}+l \hfill \\ 0\le x\le {X}_{2}\,{{{{{{\rm{and}}}}}}}\,{X}_{2}+l\le x\le h\end{array}.$$

Here, *X*_1_ and *X*_2_ are the positions of two negative domains. The SF complex-amplitude is given by

10 $${A}_{3}=	\int _{0}^{h}\frac{i{\omega }_{3}}{{n}_{3}c}{\chi }^{\left(2\right)}{A}_{1}{A}_{2}\exp \left(i\Delta {kx}\right){dx}-\int _{{X}_{1}}^{{X}_{1}+l}\frac{2i{\omega }_{3}}{{n}_{3}c}{\chi }^{\left(2\right)}{A}_{1}{A}_{2}\exp \left(i\Delta {kx}\right){dx} \\ 	 -\int _{{X}_{2}}^{{X}_{2}+l}\frac{2i{\omega }_{3}}{{n}_{3}c}{\chi }^{(2)}{A}_{1}{A}_{2}\exp \left(i\Delta {kx}\right){dx}\\=	{C}_{0}+C\exp \left(i\Delta k{X}_{1}\right)+C\exp \left(i\Delta k{X}_{2}\right)\\=	{C}_{0}+2C\cos \left(\Delta k\frac{{X}_{1}-{X}_{2}}{2}\right)\exp \left(i\Delta k\frac{{X}_{1}+{X}_{2}}{2}\right)$$

Then, one can obtain ${A}_{3}\propto \cos \left(\Delta k\frac{{X}_{1}-{X}_{2}}{2}\right)\exp \left(i\Delta k\frac{{X}_{1}+{X}_{2}}{2}\right).$

### Designing the nanodomain structure in a super-pixel

According to Eq. (2), the normalized amplitude *a* and phase *φ* of the generated SF wave in a super-pixel are

11 $$\left\{\begin{array}{c}a=\,\cos \Delta k\frac{{X}_{1}-{X}_{2}}{2}\\ \varphi=\Delta k\frac{{X}_{1}+{X}_{2}}{2}\hfill\end{array}\,{{{{{\rm{when}}}}}}\,\Delta k\frac{{X}_{1}-{X}_{2}}{2}\in \left[2m\pi -\frac{\pi }{2},\; 2m\pi+\frac{\pi }{2}\right]\right.$$

and

12 $$\left\{\begin{array}{c}a=-\!\cos \Delta k\frac{{X}_{1}-{X}_{2}}{2}\\ \varphi=\Delta k\frac{{X}_{1}+{X}_{2}}{2}+\pi \hfill\end{array}\,{{{{{\rm{when}}}}}} \; \Delta k\frac{{X}_{1}-{X}_{2}}{2}\in \left[2m\pi+\frac{\pi }{2},\; 2m\pi+\frac{3\pi }{2}\right]\right..$$

Here, *m* can be arbitrary integers. In this work, we choose Eq. (12) (i.e., the area marked by green dotted line in Fig. 1d, e) to design the hologram. For given *a* and *φ*, the values of *X*_1_ and *X*_2_ can be calculated by

13 $$\left\{\begin{array}{c}{X}_{1}=\frac{\varphi+\arccos (-a)}{\Delta k}\\ {X}_{2}=\frac{\varphi -\arccos (-a)}{\Delta k}\end{array}\right.$$

For example, in the generation of 3D spiral line, $\Delta k=1.85\,{{{{{\rm{rad}}}}}}\;{{{{{\rm{um}}}}}}^{-1}$ at an input wavelength of 840 nm. The complex-amplitude nonlinear hologram (i.e., the spatial distributions of *a* and *φ*) is calculated through the direct Fresnel transform. Then, the values of *X*_1_ and *X*_2_ are deduced according to Eq. (13).

### Designing a dynamic nonlinear hologram

Dynamic nonlinear holography is achieved by varying Δ*k* to modulate the functions of super-pixels. One can change the input wavelength, light polarization, and crystal temperature to tune Δ*k*. In Supplementary Fig. 3, we present the evolution of the SH complex-amplitude of various super-pixels when increasing the value of Δ*k* from Δ to 2Δ. In our design, the dynamic nonlinear hologram presents the complex-amplitude distributions of *H*_1_, *H*_2_, *H*_3_, …… for the reconstructions of different 3D images at $\Delta {k}_{1}$, $\Delta {k}_{2}$, $\Delta {k}_{3}$, ……, respectively. Each 3D image can be sliced into multiple 2D images at different propagation depths of light. After performing iterative Fourier transform, we can obtain the optimized Fourier holograms of these 2D images that satisfy the amplitude and phase constrain conditions (i.e., the dependence of the SH complex-amplitude on Δ*k* in Supplementary Fig. 3). Then, we add the phase of a Fresnel lens into these Fourier holograms and their coherent superposition composes the final Fresnel hologram. In this way, one can dynamically switch the output SH patterns from one nonlinear hologram by choosing the corresponding Δ*k*.

### Design of nonlinear DONN

The model is a single-layer neural network^61^. The numbers are carried by an NIR beam at 1342 nm and the output SF wavelength is 593 nm. The loss function is defined as the mean square error between the output results and the ground truth. We optimize the nonlinear hologram using the gradient descent method. In the training process, the learning rate is set as 0.1 and the batch size is 300. All the calculations are based on Tensorflow 2.6 with Python3.9.7. The database is adopted from MNIST handwritten digits^62^.

### Sample preparation

The sample is prepared by using the femtosecond laser writing technique in ref. ^58^. The domain structure is fabricated in a 5% MgO-doped LiNbO_3_ crystal. When the near-infrared femtosecond laser is focused into the crystal, the multiphoton absorption produces a localized temperature field, which leads to a strong head-to-head electric field at the laser spot. Meanwhile, the threshold field for LiNbO_3_ domain inversion significantly decreases because laser heating results in an increased ionic conductivity and therefore a reduction in domain wall pinning. When the laser-induced electric field is larger than the threshold field, the domain is poled and the sign of ${\chi }^{(2)}$ is inverted. Notably, only half of the laser-induced electric field (i.e., the –*z* component) is antiparallel to the spontaneous polarization of LiNbO_3_ crystal, which composes the effective electric field for domain poling. In this work, the femtosecond laser is focused along the –*x* axis and moves along the +*x* axis. Therefore, the domain size (about 500 nm in the *y*–*z* plane) is half of the laser spot size (about 1 μm). Generally, the length of the negative domain (along the *x* direction) is set to be the coherence length *l*_c_ of SH generation to enhance the maximal output SH intensity from a super-pixel. The super-pixel length should be the multiples of *l*_c_ for the complete amplitude modulation. It is necessary to avoid spatial overlap between two negative domains. For example, we use a type-0 SH generation configuration (i.e., *e* + *e* → *e*) for the generation of spiral line (Fig. 2). The lengths of the negative domain and super-pixel are *l*_c_ = 1.7 μm and 5*l*_c_ = 8.5 μm, respectively. It should be noted that we add a blazing grating structure into the nonlinear hologram to avoid the background SH waves.

### Experimental setup of nonlinear holography

The fundamental beam is output from a Ti:Sapphire femtosecond laser (Chameleon, Coherent), featuring a 75 fs pulse duration, an 80 MHz repetition rate, and a tunable wavelength range from 690 nm to 1050 nm. The polarization of fundamental beam is controlled by a half-wave plate. After passing through a 125 mm lens, the 80 μm-in-diameter fundamental beam is incident into the sample. The sample is set in a temperature-controlling oven. The generated SH pattern is imaged onto a camera by using an object lens (20×). Here, a short-pass filter is used to filter out the fundamental beam. By moving the object lens, the SH patterns at different positions are recorded, which compose a complete 3D image. In experiment, we use type-0 phase matching (i.e., *e* + *e* → *e*) in Figs. 2, 3e, f, 5 and 6 and type-I phase matching (*o* + *o* → *e*) in Figs. 3c, d and 4. The conversion efficiency is measured to be about 10^−6^, which can be significantly improved by increasing the hologram layers according to QPM theory.

### Supplementary information

Supplementary Information

Peer Review File

## Data Availability

The data that supports the results in this paper are available within the paper and its supplementary information files.
