# Supplementary material for: Laser nanoprinting of 3D nonlinear holograms beyond 25000 pixels-per-inch for inter-wavelength-band information processing
Source: Nat Commun. 2023 Sep 8;14:5523. doi: 10.1038/s41467-023-41350-2 (PMC10491822; doi:10.1038/s41467-023-41350-2)
Supplement: Supplementary file 1 — Supplementary Information [file 41467_2023_41350_MOESM1_ESM.pdf]

# Supplementary Information：

# Laser nanoprinting of 3D nonlinear holograms beyond 25000 pixels-per-inch for inter-wavelength-band information processing

Pengcheng Chen[1,†], Xiaoyi Xu[1,†], Tianxin Wang[1], Chao Zhou[1], Dunzhao Wei[2], Jianan Ma[1], Junjie Guo[1], Xuejing Cui[1], Xiaoyan Cheng[1], Chenzhu Xie[1], Shuang Zhang[3,4], Shining Zhu[1], Min Xiao[1,5], and Yong Zhang[1,*]

[1]National Laboratory of Solid State Microstructures, College of Engineering and Applied Sciences, School of Physics, and Collaborative Innovation Center of Advanced Microstructures, Nanjing University, Nanjing 210093, China

[2]School of Physics, Sun Yat-sen University, Guangzhou 510275, China

[3]Department of Physics, The University of Hong Kong, Hong Kong, China

[4]Department of Electrical and Electronic Engineering, University of Hong Kong, Hong Kong, China

[5]Department of Physics, University of Arkansas, Fayetteville, Arkansas 72701, USA

[†]These authors contribute equally to this work

*Corresponding author: zhangyong@nju.edu.cn

**Supplementary Note 1. Comparing the outputs from the directly-transmitted fundamental wave channel and the SH channel**

In comparison to its linear counterpart, our nonlinear hologram stores the information into the spatial distribution of nonlinear coefficients $\chi^{(2)}$, which can be only revealed in the nonlinear waves at harmonic frequencies. This can be useful in circumstances that require high security because one cannot observe the information in the directly-transmitted fundamental light. In the experiment, we use the generation of 3D spiral line as an example (Supplementary Fig. 1). After passing an 840 nm light through the nonlinear hologram, one can observe the spiral line at the SH channel but nothing at the fundamental wave channel.

**Supplementary Note 2. Dynamic nonlinear beam shaping**

We demonstrate the dynamic conversions between orbital angular momentum (OAM)[1] beams of $l = 1$, $l = 2$, and $l = 3$ for example. Here, $l$ is topological charge (TC). The nonlinear hologram has a spiral phase. The dynamic modulation is realized by tuning the input wavelength and crystal temperature to change the value of $\Delta k$. The experimental configuration is shown in Supplementary Fig. 2a. Supplementary Fig. 2b-i shows the OAM patterns at second-harmonic (SH) waves and the measurements of their TCs. First, we set the input wavelength at 910 nm and the crystal temperature at 26 °C. The output SH pattern shows a well-defined ring (Supplementary Fig. 2b). By using a cylindrical lens[2, 3], the TC is measured to be $l = 1$ (Supplementary Fig. 2c). If increasing the temperature to 440 °C (Supplementary Fig. 2d-e) or tuning the input wavelength to 820 nm (Supplementary Fig. 2f-g), one can obtain an OAM beam of $l = 2$ at SH wave. As shown in Supplementary Fig. 2h-i, the SH OAM beam of $l = 3$ present at a wavelength of 820 nm and at a temperature of 320 °C.

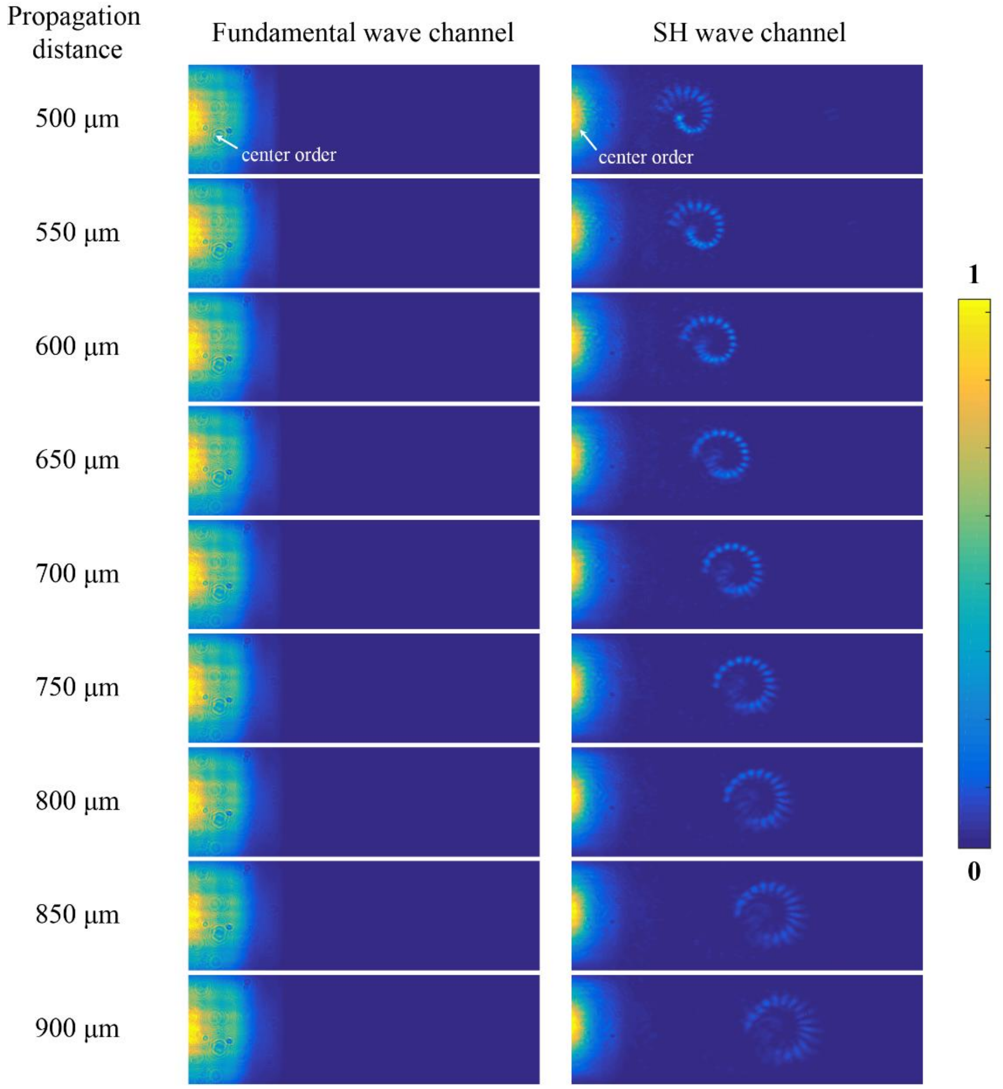


**Supplementary Fig. 1** Comparison of the directly-transmitted fundamental wave channel and the SH channel. Here, we input an 840 nm light into the nonlinear hologram.

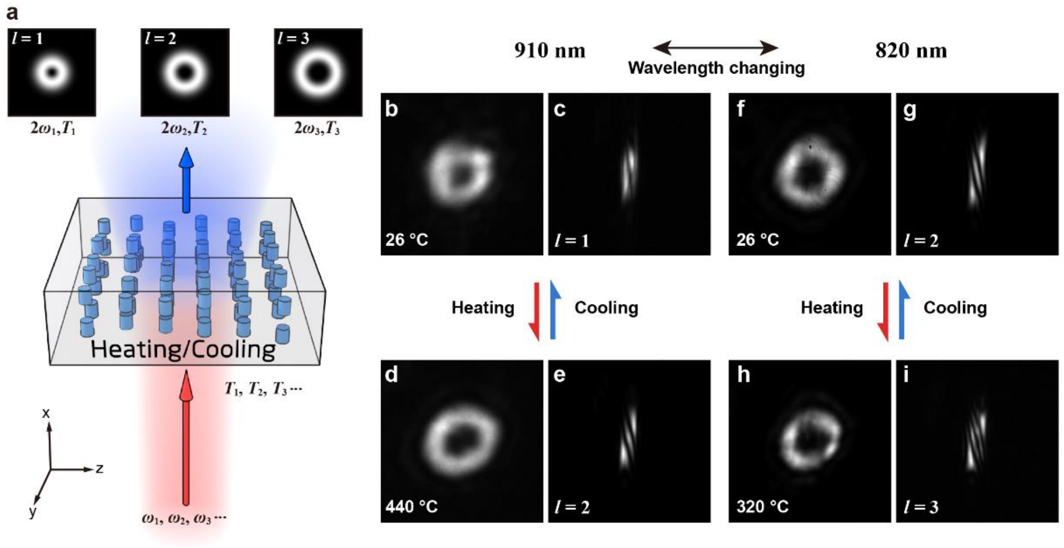


**Supplementary Fig. 2** Dynamic nonlinear beam shaping. **a** The experimental configuration. **b, d, f, and h** show the SH patterns at various experimental conditions. The corresponding TC measurements by using a cylinder lens are shown in **c, e, g,** and **i**, respectively. One can obtain the TCs by counting the numbers of dark lines.

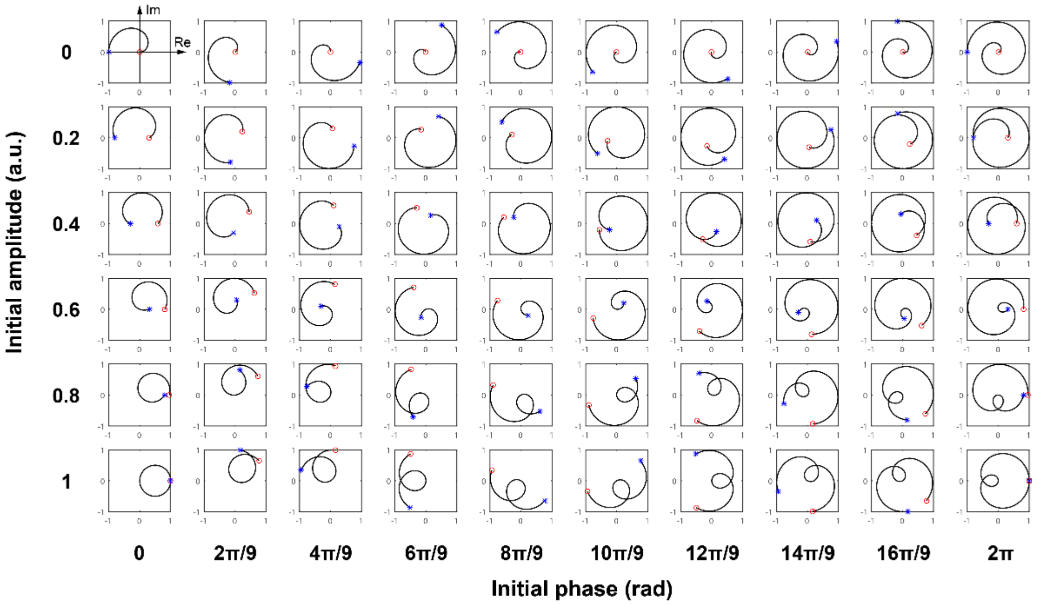


**Supplementary Fig. 3** The evolution trajectories of SH complex-amplitudes as increasing the value of $\Delta k$ from $\varDelta$ to $2\varDelta$. Each block presents a complex plane, from which one can read the normalized amplitude and phase of SH wave. The initial SH amplitudes and phases at $\Delta k = \varDelta$ are presented, which correspond to the red circles in each block. The blue asterisks represent the final state of $\Delta k = 2\varDelta$. The black lines show the evolution trajectories.

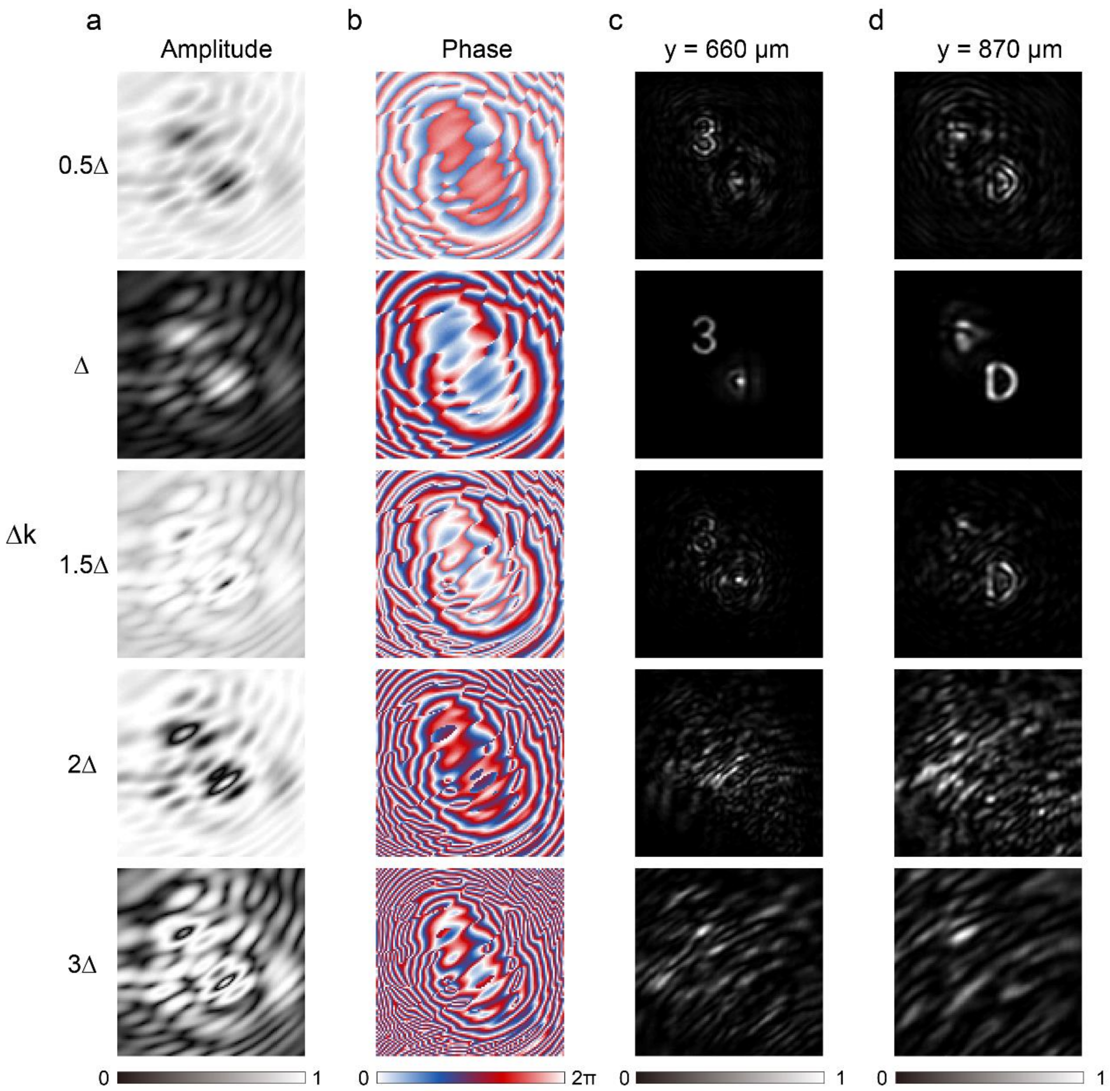


**Supplementary Fig. 4** Dependence of encryption storage on $\Delta k$. In this case, we store 3D information in the channel of $\Delta k = \Delta$ (see the second line). **a** and **b** show the amplitude and phase distributions of nonlinear hologram under different $\boldsymbol{\Delta k}$. **c** and **d** present the reconstructed 3D images. When gradually tuning $\Delta k$ away from $\Delta$, the pattern begins to blur (see the first and third lines). When $\Delta k > 2\Delta$ (see the last two lines), one can hardly distinguish the information.

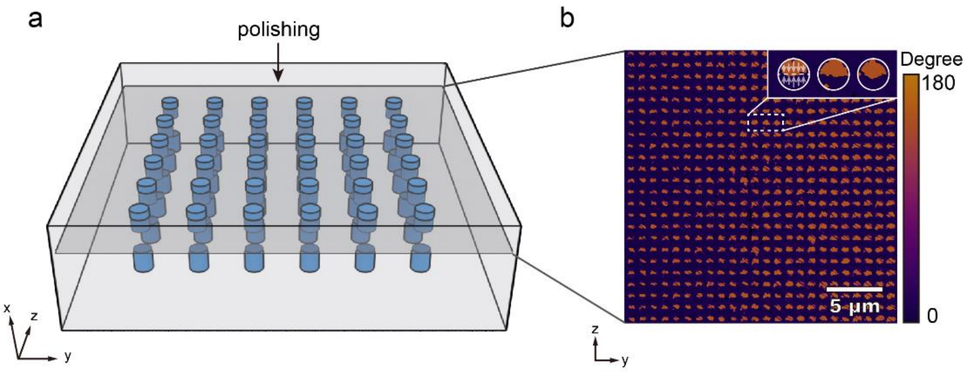


**Supplementary Fig. 5** The characterization of nonlinear hologram. **a** The domain structure is polished to the sample surface. **b** shows the piezo-response force microscopy (PFM) image of the domain structure. The inset in **b** shows that only the upper half of the laser-illuminated area (i.e., the white circle) is poled. Because the laser-induced thermoelectric field points to the center of laser spot, only the upper half is anti-parallel to the spontaneous polarization of $LiNbO_3$ crystal, composing an effective field to invert the ferroelectric domains.

**Supplementary Table 1** The data library linking the amplitude and phase of the generated SH wave with the positions of nanodomain for the experiment in Fig. 2

| Amplitude / Phase (rad) | 0 | 0.1 | 0.2 | 0.3 | 0.4 | 0.5 | 0.6 | 0.7 | 0.8 | 0.9 | 1 |
|---|---|---|---|---|---|---|---|---|---|---|---|
| 0 | 0.85 | 0.79 | 0.74 | 0.68 | 0.63 | 0.56 | 0.5 | 0.43 | 0.35 | 0.24 | 0 |
| | 2.54 | 2.59 | 2.65 | 2.71 | 2.76 | 2.82 | 2.89 | 2.96 | 3.04 | 3.14 | 3.39 |
| 0.2π | 1.19 | 1.13 | 1.08 | 1.02 | 0.96 | 0.9 | 0.84 | 0.77 | 0.69 | 0.58 | 0.34 |
| | 2.88 | 2.93 | 2.99 | 3.04 | 3.1 | 3.16 | 3.23 | 3.3 | 3.38 | 3.48 | 3.73 |
| 0.4π | 1.52 | 1.47 | 1.42 | 1.36 | 1.3 | 1.24 | 1.18 | 1.11 | 1.02 | 0.92 | 0.68 |
| | 3.22 | 3.27 | 3.33 | 3.38 | 3.44 | 3.5 | 3.57 | 3.64 | 3.72 | 3.82 | 4.07 |
| 0.6π | 1.86 | 1.81 | 1.75 | 1.7 | 1.64 | 1.58 | 1.52 | 1.45 | 1.36 | 1.26 | 1.02 |
| | 3.56 | 3.61 | 3.67 | 3.72 | 3.78 | 3.84 | 3.9 | 3.98 | 4.06 | 4.16 | 4.4 |
| 0.8π | 2.2 | 2.15 | 2.09 | 2.04 | 1.98 | 1.92 | 1.86 | 1.78 | 1.7 | 1.6 | 1.36 |
| | 3.9 | 3.95 | 4 | 4.06 | 4.12 | 4.18 | 4.24 | 4.31 | 4.4 | 4.5 | 4.74 |
| π | 2.54 | 2.49 | 2.43 | 2.38 | 2.32 | 2.26 | 2.19 | 2.12 | 2.04 | 1.94 | 1.69 |
| | 4.23 | 4.29 | 4.34 | 4.4 | 4.46 | 4.52 | 4.58 | 4.65 | 4.73 | 4.84 | 5.08 |
| 1.2π | 2.88 | 2.83 | 2.77 | 2.72 | 2.66 | 2.6 | 2.53 | 2.46 | 2.38 | 2.28 | 2.03 |
| | 4.57 | 4.63 | 4.68 | 4.74 | 4.8 | 4.86 | 4.92 | 4.99 | 5.07 | 5.18 | 5.42 |
| 1.4π | 3.22 | 3.16 | 3.11 | 3.05 | 3 | 2.94 | 2.87 | 2.8 | 2.72 | 2.61 | 2.37 |
| | 4.91 | 4.97 | 5.02 | 5.08 | 5.13 | 5.19 | 5.26 | 5.33 | 5.41 | 5.52 | 5.76 |
| 1.6π | 3.56 | 3.5 | 3.45 | 3.39 | 3.34 | 3.27 | 3.21 | 3.14 | 3.06 | 2.95 | 2.71 |
| | 5.25 | 5.31 | 5.36 | 5.42 | 5.47 | 5.53 | 5.6 | 5.67 | 5.75 | 5.86 | 6.1 |
| 1.8π | 3.9 | 3.84 | 3.79 | 3.73 | 3.67 | 3.61 | 3.55 | 3.48 | 3.4 | 3.29 | 3.05 |
| | 5.59 | 5.64 | 5.7 | 5.75 | 5.81 | 5.87 | 5.94 | 6.01 | 6.09 | 6.19 | 6.44 |
| 2π | 4.23 | 4.18 | 4.13 | 4.07 | 4.01 | 3.95 | 3.89 | 3.82 | 3.73 | 3.63 | 3.39 |
| | 5.93 | 5.98 | 6.04 | 6.09 | 6.15 | 6.21 | 6.28 | 6.35 | 6.43 | 6.53 | 6.78 |

(shaded) represent $X_1$ (μm), (unshaded) represent $X_2$ (μm)

**Supplementary References**


1. Allen, L., Beijersbergen, M.W., Spreeuw, R.J. & Woerdman, J.P. Orbital angular momentum of light and the transformation of Laguerre-Gaussian laser modes. *Phys Rev A* **45**, 8185-8189 (1992).
2. Denisenko, V. et al. Determination of topological charges of polychromatic optical vortices. *Opt Express* **17**, 23374-23379 (2009).
3. Fang, X. et al. Examining second-harmonic generation of high-order Laguerre-Gaussian modes through a single cylindrical lens. *Opt Lett* **42**, 4387-4390 (2017).
